# Sex differences in drugs: the development of a comprehensive knowledge base to improve gender awareness prescribing

**DOI:** 10.1186/s13293-017-0155-5

**Published:** 2017-10-24

**Authors:** Linnéa Karlsson Lind, Mia von Euler, Seher Korkmaz, Karin Schenck Gustafsson

**Affiliations:** 1 Department of E-health and Strategic IT, Health and Medical Care Administration, Stockholm County Council, Box 17533, 118 91 Stockholm, Sweden; 2 Department of Clinical Science and Education, Södersjukhuset, Karolinska Institutet, Stockholm, Sweden; 3 Department of Clinical Pharmacology, Karolinska University Hospital, Stockholm, Sweden; 4 Department of Medicine, Division of Clinical Pharmacology, Karolinska Institutet, Stockholm, Sweden; 5 Department of Health Care Development, Health and Medical Care Administration, Stockholm County Council, Stockholm, Sweden; 6 Department of Medicine, Centre for Gender Medicine, Karolinska Institutet, Stockholm, Sweden; 7 Department of Medicine, Cardiac Unit, Karolinska University Hospital, Stockholm, Sweden

## Background

Equity in healthcare implies that patients should receive healthcare based on their actual needs. This means that caregivers must have knowledge of, and allow for, biological sex differences as well as sex-specific needs, which otherwise may lead to incorrect diagnoses and lack of treatment response [1, 2]. According to the World Health Organization, rational use of medicines requires that “patients receive medications appropriate to their medical needs, in doses meeting their own individual requirements, for an adequate period of time and to the lowest costs to them and to the community” [3]. Thus, rational drug use requires knowledge of differences in drug effect in men and women, at different ages, with varying co-morbidity, and of different ethnicity [4, 5].

Differences in use of medical drugs between men and women have been reported with examples from many therapeutic areas [4, 6–8]. Also, several studies have shown that adverse events are more frequent in women than men [9–11]. Some of these differences can be attributed to differences in physiology and morbidity between men and women, as well as in pharmacokinetics [12–14] and pharmacodynamics [15]. However, other studies have presented inexplicable sex and gender differences [16, 17]. Differences may be explained by a combination of biological (sex) and socio-cultural-ethnic factors (gender) or only one of these.

However, information on sex and gender aspects of medical drugs is difficult to find. Information sources like Physicians’ Desk Reference offer some information without any references to the evidence; there are hardly any recommendations on how to manage drug treatment in men/boys or women/girls. In a qualitative study, primary care physicians expressed a lack of knowledge on how to amend for sex and gender when prescribing drugs [18]. To meet the demand of information and knowledge on sex differences, we initiated a project on a comprehensive knowledge base which received funding from Swedish Government via Swedish Association of Local Authorities and Regions (SALAR), Karolinska Institutet, Center for Gender Medicine, in collaboration with Stockholm County Council. The project was also a part of the local program for rational drug use in Stockholm County (www.janusinfo.se). The web-based knowledge base *Janusmed Sex and Gender* is a decision support for drug prescribing and dose recommendations where the sex-specific needs are considered. The work with the knowledge base started, and the first version was published in September 2013 and has subsequently been developed in a stepwise manner. Extensive searches following a standardized operational procedure are carried out together with free searches; and the texts are discussed in cross professional and interdisciplinary groups before publications.

## Development of the knowledge base

**Fig. 1:**
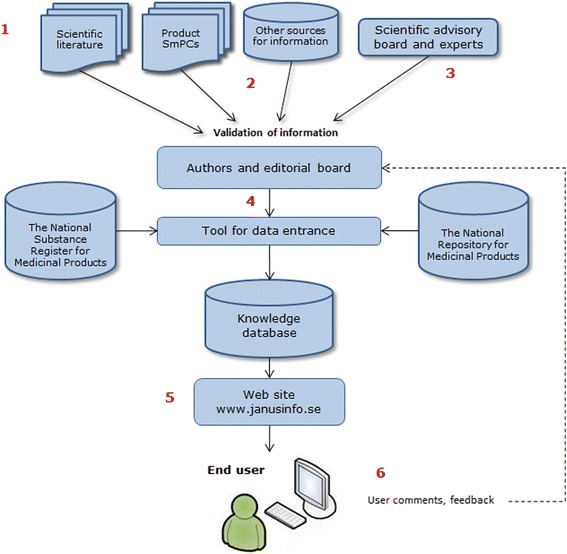
Workflow for producing and maintaining the knowledge base Janusmed Sex and Gender. The numbers refer to each step described in the “Methods” section

Working procedures for Janusmed Sex and Gender are adopted from a comprehensive concept developed for pharmacological knowledge bases [19]. A knowledge base contains aggregate medical knowledge and no individual patient data. Figure 1 shows the working process and each step is explained in more detail below.

### Literature searches

Standard instructions for literature searches have been developed to improve work consistency. Systematic searches of scientific publications are performed in PubMed using combinations of the terms “gender,” “sex,” “sex factors,” “sex differences,” “gender differences,” “female,” “male,” “women,” “men,” and individual substance names (see Additional file 1). Publications are selected based on information regarding any of the following aspects: pharmacokinetics, dosing, effects, and adverse effects. No time limitation for publication year is used. Publications with a sex analysis of the results or presenting results data divided by patient’s sex are eligible for validation. Gender differences are more difficult to study in this area, and the information is indeed very limited. Some examples of gender differences might be seen in the utilization of drugs, for example, the antiobesity drug orlistat, which is almost three times more commonly used by women in Sweden [20]. However, overweight is more common in men [21]. Gender is often used to describe any difference between males and females, cultural/hormonal, or genetic/hormonal because the border between sex and gender is often very diffuse [22]. Because we consider most of the differences such as pharmacokinetics depend on the biology, we have used the word “sex.”

We exclude information from case reports, letters, non-full text articles, withdrawn studies, and trials on animals. The rationale underlying these exclusion criteria is to focus on higher quality scientific work and homogenize the included population. Studies in which only persons of one sex are excluded are used after special consideration and only if other data is not available. For example, if studies in women are missing despite that the prevalence of the disease is similar or even more common, then single sex studies are motivated. In drugs, used specifically in one sex, for example oral contraceptives for women or treatment for prostate cancer in men, no comparison or search for sex differences is made as this is not relevant.

Other sources of information include reference textbooks (such as Martindale and Dollery [23–25]), summary of product characteristics for individual preparations in Sweden and USA, and clinical review sections of new drug approval packages [26]. Search results from the literature are read, sorted, and, if needed, discussed with the working group.

Regarding selection of drugs to include into the knowledge base, we choose to focus on medicinal substances included in the local Drug and Therapeutic Committees recommended drug list, “Wise List,” covering treatment of common conditions in primary and outpatient care. The Wise List has shown to have high adherence and impact on prescribing [27]. However, our knowledge base also covers many other commonly used substances not included in the Wise List.

### Epidemiological data extraction

Data on drugs dispensed to all males and females in Sweden are added to each text document in the Swedish version. Data are derived from the Statistical Database on Pharmaceuticals held by the Swedish National Board of Health and Welfare, containing dispensed prescriptions to all Swedish citizens at Swedish pharmacies since 2006 [20]. For every substance, information on number of patients, patient’s sex and age, and the total number of dispensed prescriptions in the last year is extracted. Non-prescription drugs, i.e., over-the-counter drugs and requisition drugs used only in hospital, are not registered on user, and therefore, sex-divided dispensing data of these drugs are not available.

### Production of text documents

Findings from the literature searches adjudicated to be of relevance are summarized for each substance into a text document. All documents are structured with following subtitles: (i) “Summary” with general recommendations, (ii) “Additional Information,” containing detailed information on pharmacokinetics, dosing, effects, and adverse effects. In some cases, information on potential for teratogenicity and interaction with sex hormones are also presented; (iii) “Reference list,” with linkage to PubMed to enable quick access to the original publication. (iv) Data on prescription drug dispensing is available for users on the Swedish version.

The text documents are produced by pharmacists and physicians having their residency in clinical pharmacology. The texts are written from a general practitioner’s perspective with short and concise assessments intended for use at point-of-care. To further improve readability, the working group has created a list of standard phrases and expressions used for harmonization of the text documents. Drugs that are closely related, such as a class of substances, can have a generic core text that summarizes information about the whole class. This reduces the time for entering the information and the size of the database. This has been done for example anticholinergic drugs used for urgency incontinence.

Some substances are available in several pharmaceutical formulations which can have local or systemic effects. Based on this, one substance could have been divided into two text documents, one covering its local effects and another covering its systemic effects.

### Analysis of clinical relevance

Each text document undergoes a careful validation by members of the working group consisting of clinical experts in gender medicine, clinical pharmacology, neurology, cardiology and pharmacy. When needed, expertise from the local Drug and Therapeutic Committee or experts from other disciplines are consulted in finalizing the documents before publication. Occasionally experts at the Swedish Medical Product Agency (MPA), the US Food and Drug Administration (FDA) or the responsible pharmaceutical drug company are consulted.

The text documents are assigned to one of four classification categories based on the amount of evidence found in the literature searches (Table 1). For drugs in class A, we have found evidence of no sex differences in our literature searches, or we have found publications reporting non-clinically relevant sex differences. Drugs in class B are lacking information on sex differences. In these cases, we have performed literature searches but have not found any publications reporting sex differences. For drugs in class C, clinically relevant sex differences influencing treatment have been shown for certain patient groups, such as pregnant women or the elderly, and therefore, the recommendations cannot be generalized to apply to all men or all women. The category C! contains drugs with clinically relevant sex differences influencing treatment. The classification system has been developed by the authors in cooperation with the clinical experts, technicians, and colleagues.

**Table 1:** Classification used in the knowledge base Janusmed Sex and Gender

Class A	
No sex differences shown or no clinically relevant sex differences	
Class B	
Data on sex differences are lacking	
Class C	
Clinically relevant sex differences in some patient populations	
Class C!	
Clinically relevant sex differences	

### Publication process

Information and text documents are stored in a database by using an editorial tool allowing access for several simultaneous users. The database is linked to The National Substance Register and The National Repository for Medical Products covering all drugs in Sweden, making it possible for the end user to search on both substance names and product names. Linkage to the registers is possible by using the unique substance identifiers ID number in the National Substance Register (NSL-id) and Anatomical Therapeutic Chemical classification codes (ATC code). The linkage function makes it possible to exclude drugs containing the same substance, but with different formulations or used in different strengths. The National Repository for Medicinal Products are also used to detect new products entered to the Swedish market. Changed or missing ATC codes are also identified by the editorial tool.

### Distribution

**Fig. 2:**
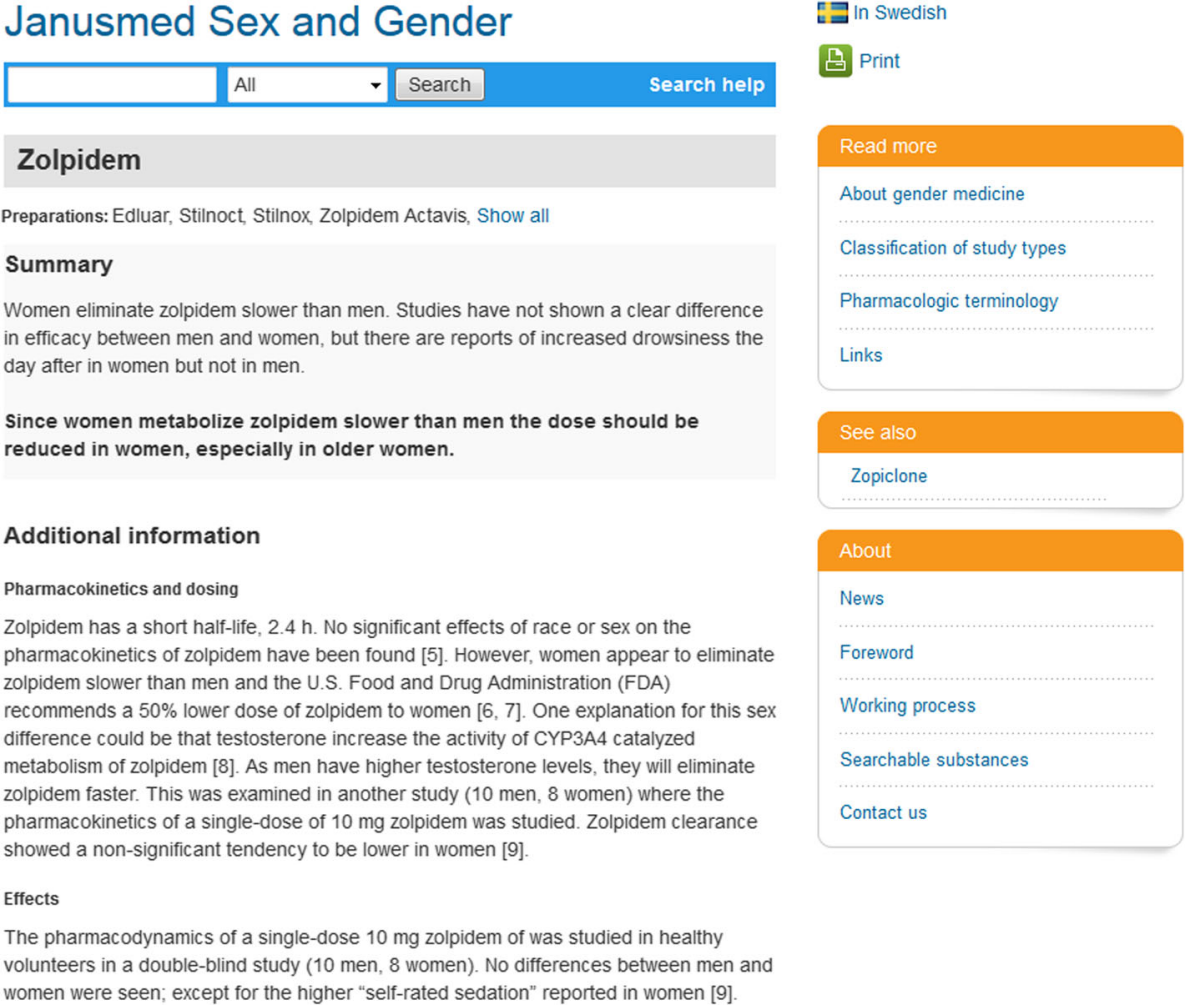
Example of a text document from the knowledge base Janusmed Sex and Gender

Access to the knowledge base is enabled via the Swedish website Janusinfo (www.janusinfo.se, subsection “*Janusmed kön och genus*”), an independent site from the Drug and Therapeutic Committee in Stockholm County Council. Since May 2016, the knowledge base is fully available in English (www.janusinfo.se/genus/in-english). The knowledge base is based on substances which are presented in separate documents (Fig. 2). Searches can be performed by substance name, ATC code or product name on the Swedish market. For medical products containing a combination of two or more substances, searches will result in one hit per substance.

### Updates

Production and update strategy, based upon our experiences from other knowledge bases, has three tiers. Rapid/immediate updates are carried out when critical information is reported on any of the drugs already included in the knowledge base. Second tier consists of drugs recently introduced to the Swedish market. The third tier, consists of a systematic review of existing material for improvements in text, terminology, and literature references. Life cycle of information content is set to 4 years, i.e., all documents in a knowledge base are set to be revised at least every fourth year for higher quality and accuracy of the content. On the first page of our website, relevant news about publications of sex differences in drugs or other aspects of gender medicine are commented.

## Use of the knowledge base

### Coverage and distribution of classification

To date, the knowledge base contains information on almost 300 substances within several therapeutic areas, representing substances from all 14 main groups in the ATC system. Approximately 70% of the recommended substances for treatment in primary care, specialized outpatient care and inpatient care in Stockholm region (The Wise List [28]) are covered. Additionally, the knowledge base includes several other commonly prescribed medicines in Sweden. The knowledge base is continuously expanding to cover more commonly used substances.

**Fig. 3:**
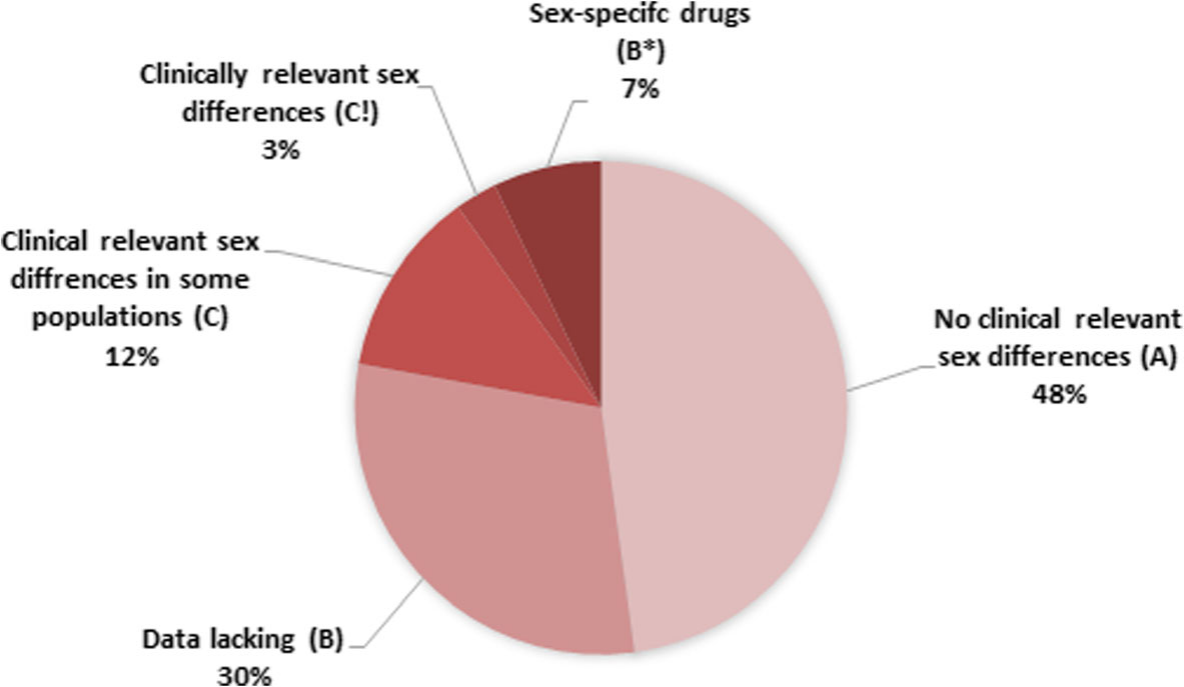
Distribution of classifications categories among published text documents in the knowledge base

Figure 3 shows the percentage of documents in each of the four classification categories. Most of the substances are categorized as class A. Sex-specific drugs are categorized as a separate category (B*).

### Search statistics and users of the knowledge base

Usage of the knowledge base is continuously evaluated via a basic statistical function in the editorial tool identifying total number of searches over time and documents. It is also possible to identify the geographically distribution of the end users and words searched.

Between January and December 2016, the number of searches in the knowledge base was around 300 per month. During the first months after the launch, the average number of searches per month was over 500. From 2014 onwards, the numbers have been around 250–300 per month. Table 2 shows the substances that generated most searches in 2016. These substances represent some of the most commonly used substances in Sweden.

**Table 2:** Top searches in Janusmed Sex and Gender

Substance	Number of searches (*n*)^a^Jan-Dec 2016
Codeine	558
Paracetamol (acetaminophen)	441
Ibuprofen	277
Diclofenac	256
Sertraline	244
Citalopram	241
Methylphenidate	233
Enalapril	231
Zolpidem	227
Aspirin–low dose	223
Etoricoxib	220
Amlodipine	217
Carbamazepine	216
Warfarin	214
Fosphenytoin	211
Pregabalin	210
Phenoxymethylpenicillin	208
Apixaban	207
Chlortalidone	206
Escitalopram	205

^a^The number of searches refers to the total amount of retrievals for a substance including all drug forms and combination products

Even though the knowledge base is published on a regional website from the capital of Sweden, the Stockholm County Council, majority of the users (56%) during 2016 were from other cities across Sweden. In total, 6.3% of the users were from other countries, mainly from Italy and France.

A pilot pop-up questionnaire on the website was carried out between October 19 and December 20 in 2015, with the aim to estimate frequency of use and user satisfaction. The response rate was 69% (*n* = 50), and the users were mainly pharmacists, resident physicians, and nurses (80% women). Many of the users (38%) reported that they did not know that the knowledge base existed. However, a high proportion was willing to recommend the knowledge base to a friend or colleague (65%). Results from the questionnaire where the responders are asked about their opinions on a scale of 1–7 (1 lowest––do not agree and 7 highest––completely agree) are presented in Table 3. Due to small sample size, no statistical analysis is performed.

**Table 3:** Results from the pop-up questionnaire

Statement: “The knowledge base Janusmed Sex and Gender…”	Average score (1–7)	Number of responders
“...is supportive in practicing my profession.”	4.71	14
“…provided me with knowledge.”	5.06	17
“...will help increasing patient safety.”	5.71	14
“…gave me insights and thoughts on gender- aware prescribing.”	5.11	14

### Strengths and limitations

As far as we know, this is the first initiative to capture evidence-based medical knowledge on sex and gender aspects on drug treatment into a searchable tool. The knowledge base summarizes the evidence of sex differences for individual drugs, making it useful when selecting proper drugs for an individual patient. Nevertheless, information on sex differences are lacking for a third of the evaluated substances in the knowledge base. A large proportion of published studies are lacking sex-specific analysis or sex-stratified results data. Data submitted to the regulatory authorities by the pharmaceutical companies to support their request for approval is rarely publicly available, which complicates assessing the validity on information.

Revealed sex differences, especially pharmacokinetic sex differences, mostly turn out to be of minor significance and are often considered to be of no clinical importance. Pharmacokinetic sex differences rarely lead to changes in dose recommendations. Often, pharmacokinetic properties are adjusted for weight whereas dosing is seldom. However, for some substances, we have found evidence of pharmacokinetic sex differences leading to dose adjustments, for example, zolpidem, methylphenidate, and dabigatran. Studies sometimes show conflicting results whether there are sex differences or not for a specific substance. This could often be explained by differences in study design and setting. Many studies have inadequate representation of women and men in the study population. Pharmacokinetic studies usually have a very limited number of participants [29]. Many pharmacokinetic studies weight-adjust the findings before presentation and conclude that there are no differences between pharmacokinetic properties in men and women. However, as dosing usually is not weight-adjusted in adults, it is sometimes difficult to tell if the dosing should be adjusted when body size usually differs between men and women.

Data on dispensed prescription drugs by either sex is used as an indicator of possible unequal drug treatment between men and women. A cross-sectional study of drug utilization in men and women in Sweden showed large differences in prevalence and incidence of dispensed drugs. Some of these differences are related to differences in disease prevalence between men and women or to biological differences. However, other differences cannot be explained on medical grounds and may indicate unequal drug treatment between men and women [24].

There are some limitations related to our literature search strategy. We are aware of that the lack of Medical Subject Headings (MeSH terms) in PubMed and standardized terminology to describe sex differences may have resulted in missing some studies. We acknowledge that the content of the studies is limited to what authors have reported or presented in their studies, which may not reflect the exact results of each study. These large variations in reporting sex-stratified results underline the need for reporting. Previously, attempts to develop special search tools to validate sex and gender-specific health data have been performed in order to facilitate the literature searches [22]. Sometimes “sex differences” are named “gender differences” in scientific literature, especially in cardiovascular publications. Gender differences, as opposed to sex differences, are mostly difficult to measure, for example, for under or overtreatment. However, both sociocultural and biological factors are important for men and women’s health and for understanding the differential development of diseases in men and women [30]. Most of the differences presented in the knowledge base depend on the biology, but some gender differences are also seen, and therefore, the name of the knowledge base contains both the terms “sex” and “gender.”

There are many ways to improve readability for the end user. Important information should be easily available and short and concise. Therefore, the most important information should be provided instantly, while background information and references will be available when needed [19]. Applying a system of classifying the drugs in this knowledge base is challenging but is necessary if used in electronic health records. However, classification is a simplification and could contribute to loss of information.

The information in the knowledge base is fully available in English and therefore useful for non-Swedish users. The documents are currently linked to the Swedish medical products register, which may differ in drug assortment compared with other countries. However, it is possible to link the knowledge base to other country-specific medical product registers with little effort. Also, the list of recommended drugs in Sweden according to national and international guidelines and recommendations might different in other countries.

The value of the Janusmed Sex and Gender in clinical practice remains to be evaluated, as well as identifying areas for improvement. Small-scale pilot studies for proof of concept are considered as important in the design of optimal interventions [31]. We intend to measure user satisfaction with a qualitative approach that give input on both the technical solutions as well as the physicians’ attitudes to the medical content and acceptance in clinical work. Evaluations of the actual improvement of patient safety and effects on patient outcome are more difficult to perform.

Currently, the Janusmed Sex and Gender is only available on the web. To develop the presentation into an electronic health record (EHR) system would probably make the information even more useful. If used from an EHR, the physician will receive automatically alerts when prescribing drugs inappropriately to men and women. Janusmed Sex and Gender is equipped for linkage to a country––specific medical product register, providing the possibility of integration into any EHR, supporting alerts and gender awareness prescribing. Combining drug information with patient-level data would most likely produce the optimal benefit for the patient [19]. Medical information of various types can be available from many different sources. Clinical decision support systems (CDSS) with underlying knowledge bases help physicians make decisions and improve quality of healthcare. CDSS are also shown to be effective in supporting physicians in rational drug prescribing [19].

## Conclusions

This article presents a novel knowledge base on potential sex differences of drug treatment as a unique source of evidence-based information. Distribution and dissemination of evidence-based medical information on sex and gender differences should have a clear role in promoting gender-aware drug prescribing. This knowledge base has potential to be a valuable resource for prescribing physicians. Planned future work and improvements will include integrating the knowledge base with the electronic health record such that information can be delivered in a context-appropriate manner. Furthermore, we hope that an increased awareness of this topic increases the demand for accurate sex and gender analysis when drugs are evaluated.

## Additional file

Additional file 1: Supplementary material (DOCX 15 kb)

## Abbreviations

ATC: Anatomical Therapeutic Chemical classification
CDSS: Clinical decision support systems
EHR: Electronic health record
FDA: Food and Drug Administration
MPA: Medical Product Agency
NSL: National Substance Register
SALAR: Swedish Association of Local Authorities and Regions
