# Correction to: Sex differences in drugs: the development of a comprehensive knowledge base to improve gender awareness prescribing

**DOI:** 10.1186/s13293-017-0160-8

**Published:** 2018-01-18

**Authors:** Linnéa Karlsson Lind, Mia von Euler, Seher Korkmaz, Karin Schenck-Gustafsson

**Affiliations:** 1 Department of E-health and Strategic IT, Health and Medical Care Administration, Stockholm County Council, Box 17533, 118 91 Stockholm, Sweden; 2 Department of Clinical Science and Education, Södersjukhuset, Karolinska Institutet, Stockholm, Sweden; 3 Department of Clinical Pharmacology, Karolinska University Hospital, Stockholm, Sweden; 4 Department of Medicine, Division of Clinical Pharmacology, Karolinska Institutet, Stockholm, Sweden; 5 Department of Health Care Development, Health and Medical Care Administration, Stockholm County Council, Stockholm, Sweden; 6 Department of Medicine, Centre for Gender Medicine, Karolinska Institutet, Stockholm, Sweden; 7 Department of Medicine, Cardiac Unit, Karolinska University Hospital, Stockholm, Sweden

## Correction

Unfortunately, after publication of this article [1], two errors were noticed. The names of Linnéa Karlsson Lind and Karin Schenck-Gustafsson were formatted incorrectly, attributing incorrect elements to the Given and Family names.

Further to this, a reference in Fig. 1 was missing. The line reading, “Fig. 1 shows the working process and each step is explained in more detail below” should instead read, “Fig. 1, modified from Nörby et al. [2], shows the working process and each step is explained in more detail below”.

The figure caption for Fig. 1 should, accordingly, read, “Fig. 1 Workflow for producing and maintaining the knowledge base Janusmed Sex and Gender (modified from Nörby et al. [2]).”

Note that reference 2 in this correction corresponds to reference 20 in the original article. The original article has also been updated to correct all aforementioned errors.
