# Supplementary material for: Sex differences in drugs: the development of a comprehensive knowledge base to improve gender awareness prescribing
Source: Biol Sex Differ. 2017 Oct 24;8:32. doi: 10.1186/s13293-017-0155-5 (PMC5655861; doi:10.1186/s13293-017-0155-5)
Supplement: Supplementary file 1 — Supplementary material (DOCX 15 kb) [file 13293_2017_155_MOESM1_ESM.docx]

# Appendix

## Search strategy in PubMed

Step 1

Wide search

("Sex Factors"[Mesh] OR "Sex Characteristics"[Mesh] OR "gender difference*" OR “sex difference*” OR “based on sex” OR “based on gender” OR gender OR sex) AND *substance name*

If too many hits or many non-relevant hits, go to Step 2.

Step 2

Limit the search by seleting following filters: Species: Humans and Languages: English. Filter by selecting Article type. If still too many hits or many non-relevant hits, go to Step 3.

Step 3

Narrow search

("Sex Factors"[Mesh] OR "Sex Characteristics"[Mesh] OR sex[Title] OR gender[Title] OR "sex difference*" OR "gender difference*" OR “based on sex” OR “based on gender”) AND substance name

If nothing is found, go to Step 4.

Step 4

Specify the search, example:

(gender OR sex) AND pharmacokinetics AND substance name
